# The therapeutic potential of phytochemicals in morphine tolerance: targeting microglia-mediated neuroinflammation

**DOI:** 10.3389/fphar.2025.1669257

**Published:** 2025-11-06

**Authors:** Ruizhen Shi, Tingting Jin, Weilong Xu, Yongxin Liang

**Affiliations:** 1 Department of Anesthesiology, Women and Children’s Hospital, Peking University People’s Hospital, Qingdao University, Qingdao, Shandong, China; 2 Qingdao Medical College, Qingdao University, Qingdao, Shandong, China; 3 Department of Anesthesiology, The Affiliated Hospital of Qingdao University, Qingdao, Shandong, China

**Keywords:** morphine tolerance, microglia, neuroinflammation, phytochemicals, therapeutic potential

## Abstract

Morphine remains a first-line analgesic for both acute and chronic pain. However, its prolonged use often results in the development of tolerance, diminishing its analgesic efficacy and limiting its long-term clinical utility. Emerging evidence highlights the pivotal role of microglial activation in the central nervous system as a key contributor to morphine tolerance. Phytochemicals, natural metabolites derived from plants, have garnered attention for their multi-target mechanisms, low toxicity, and broad biological activities, positioning them as promising candidates for mitigating morphine tolerance. This review systematically explores the key receptors and signaling pathways involved in microglial activation during morphine tolerance, and elucidates how various phytochemicals modulate these pathways to attenuate tolerance. Furthermore, it discusses the translational challenges associated with phytochemical-based interventions and outlines future directions for their clinical application. The aim is to provide a theoretical framework to support translational research and the development of novel adjunct therapies for opioid analgesia.

## 1 Introduction

Morphine, a classic opioid analgesic, remains the cornerstone drug for treating moderate to severe pain. However, its prolonged use is associated with a major and challenging side effect—morphine tolerance (Bekhit, 2010). Morphine tolerance refers to the phenomenon in which patients must gradually increase the drug dosage to maintain the initial level of analgesia. This not only diminishes the analgesic efficacy but also increases the risk of adverse effects, including nausea, vomiting, respiratory depression, and addiction (Ing Lorenzini et al., 2022). Consequently, a deeper understanding of morphine tolerance mechanisms and the discovery of effective countermeasures are urgently needed in pain pharmacology.

Traditionally, research on the mechanisms of morphine tolerance has primarily focused on neurons, particularly involving μ-opioid receptor (MOR) desensitization, internalization dysfunction, and compensatory upregulation of intracellular cyclic adenosine monophosphate (cAMP) signaling and other adaptive neuronal changes (Williams et al., 2013; Zhang et al., 2023; Lim et al., 2005). However, accumulating evidence suggests that innate immune cells in the central nervous system (CNS), specifically microglia, play a crucial role in the development of morphine tolerance (Du et al., 2020; Zhang et al., 2024). As the first line of immune defense in the CNS, microglia mediate neuroinflammatory responses by releasing pro-inflammatory cytokines such as interleukin-1β (IL-1β), tumor necrosis factor-α (TNF-α), and interleukin-6 (IL-6), disrupting neuronal homeostasis and accelerating the development of morphine tolerance (Shen et al., 2012).

Currently, no drugs have been approved for clinical use specifically to prevent or reverse morphine tolerance. Conventional strategies, such as increasing opioid doses or rotating opioids, have limited efficacy and are associated with significant risks. Therefore, there is an urgent need to develop new adjunctive therapies. In this context, phytochemicals, with their wide availability, structural diversity, favorable safety profiles, and capacity to regulate multiple biological targets, have emerged as valuable resources for novel drug development (Patridge et al., 2016). Preclinical evidence indicates that various phytochemicals, including flavonoids, alkaloids, and phenolics, can effectively inhibit the abnormal activation of microglia, thereby alleviating morphine tolerance in rodent models, demonstrating their great potential for further development.

Here we summarize the critical role of microglia-mediated neuroinflammation in morphine tolerance, with a particular focus on summarizing recent research on phytochemicals that modulate microglial function to alleviate morphine tolerance. Additionally, the review discusses the limitations of current research and the challenges for clinical translation, aiming to provide new perspectives on the application of phytochemicals in the treatment of morphine tolerance.

## 2 Literature search methodology

A systematic search was conducted using PubMed and Web of Science, with a cutoff date of June 2025. The search strategy included keyword combinations such as “morphine tolerance,” “microglia,” “inflammation,” “natural compounds,” and “phytochemicals” to ensure the relevance and accuracy of the results. Studies included in this review were required to investigate the role of microglia in the mechanisms of morphine tolerance or to explore how phytochemicals alleviate morphine tolerance by targeting microglia. All included studies were published in peer-reviewed journals.

## 3 Microglia and morphine tolerance

Microglia, the resident immune cells of the CNS, are critical for maintaining neuroenvironmental homeostasis (Azam et al., 2021). In both humans and rodents, microglia originate from erythro-myeloid progenitors (EMPs) in the yolk sac, although the timing and processes of their development differ between species. In humans, EMPs emerge between gestational weeks 4 and 5, migrate from the meninges and the ventricular zone to the developing brain, and mature during later pregnancy stages. In rodents (e.g., mice), EMPs appear between embryonic days 7.5 and 9, colonize the brain via trans-tissue and transvascular routes, and rapidly differentiate into mature microglia (Chen et al., 2025). These developmental differences may contribute to variations in microglial function and their response mechanisms across species, thereby influencing their roles in disease.

Under normal physiological conditions, microglia are in a resting state, primarily conducting immune surveillance and providing neuroprotection through their elongated processes. Upon damage, infection, or inflammation in the nervous system, microglia rapidly become activated, with increased cell body size and shortened processes, adopting an ameboid morphology. Based on their activation states, microglia are typically classified into two types: classical pro-inflammatory activation (M1) and alternative anti-inflammatory activation (M2) (Depp et al., 2025). Although advanced techniques such as single-cell RNA sequencing (scRNA-seq) have revealed the complexity and heterogeneity of microglial phenotypes, far beyond the traditional M1/M2 dichotomy, this classification remains widely used to describe their functional states. M1 microglia, through the secretion of pro-inflammatory cytokines (e.g., TNF-α, IL-1β, and IL-6), activate neuroinflammatory responses, which generally have a detrimental role in morphine tolerance. In contrast, M2 microglia promote tissue repair and neuroprotection through the secretion of anti-inflammatory factors (e.g., IL-10, TGF-β), which help alleviate morphine tolerance (Tu et al., 2021). Therefore, modulating the balance between M1 and M2 microglia is considered a potential therapeutic strategy for reducing morphine tolerance.

Moreover, microglia exhibit significant sex dimorphism in their functions, which may contribute to the sex differences observed in morphine tolerance. In rodent models, female mice are more susceptible to developing morphine tolerance than male mice (Gabel et al., 2023). Clinical studies also show that female patients typically require approximately 30% higher doses of morphine than male patients to achieve comparable analgesic effects (Averitt et al., 2019; Cepeda and Carr, 2003). However, existing studies on morphine tolerance have predominantly focused on male animal models. Future research on therapeutic strategies for morphine tolerance should incorporate sex as a variable to comprehensively assess drug efficacy and further elucidate the underlying mechanisms.

## 4 Microglia-mediated neuroinflammation in morphine tolerance

### 4.1 Microglial receptors in morphine tolerance

Microglia are the primary immune cells mediating the brain’s inflammatory response. They express various receptors on both their surface and intracellular compartments, which are implicated in activation, migration, and phagocytosis. Morphine directly or indirectly activates these receptors, triggering multiple intracellular signaling pathways that result in the release of numerous cytokines, thereby promoting the development of morphine tolerance.

#### 4.1.1 μ-opioid receptor (MOR)

The expression of MOR encoded by the Oprm1 gene in microglia remains controversial. Several studies provide strong evidence for the presence and function of MOR in microglia. Maduna et al. detected Oprm1 mRNA and MOR protein in microglia isolated from both rodents and humans (Maduna et al., 2018), directly confirming its existence. Further experiments by Reiss et al. demonstrated that specific knockout of MOR in microglia significantly delayed the development of morphine tolerance, with this effect consistently observed in both male and female mice, suggesting that microglial MOR plays a crucial role in regulating morphine tolerance (Reiss et al., 2022). Additionally, other studies have shown that morphine can activate a series of signaling pathways through MOR in microglia, particularly the MOR-protein kinase C epsilon (PKCε)-protein kinase B (Akt)-Extracellular signal-regulated kinase 1/2 (ERK1/2) signaling axis, leading to upregulation of inducible nitric oxide synthase (iNOS) and enhanced nuclear factor-kappa B (NF-κB) activity, which in turn promotes the release of nitric oxide (NO) and pro-inflammatory cytokines such as IL-1β, IL-6, and TNF-α (Merighi et al., 2013; Gessi et al., 2016). These studies collectively support the expression of MOR in microglia and its involvement in morphine tolerance.

However, other studies have reached opposing conclusions. Kao et al. found that morphine still induced microglial activation in global MOR knockout mice (Kao et al., 2012). Corder et al. used high-sensitivity RNA sequencing and *in situ* hybridization techniques but detected no significant MOR expression in microglia, raising concerns regarding the accuracy and sensitivity of previous detection methods (Corder et al., 2017).

Despite the contradictory results in the existing literature, the majority of evidence supports the expression of MOR in microglia and its role in regulating morphine tolerance. However, to fully resolve this controversy and clarify the exact role of microglial MOR in morphine tolerance, future studies should employ more advanced and precise techniques. For example, scRNA-seq can reveal gene expression profiles of different microglial subpopulations, which will aid in accurately detecting MOR expression and addressing the confounding effects of microglial heterogeneity.

#### 4.1.2 Toll-like receptors (TLRs)

TLRs are a class of glycoproteins located on the cell membrane, and studies have shown that microglia express all known TLRs (from TLR1 to TLR9) (Olson and Miller, 2004). Among these, TLR4 has been the most extensively studied in the context of morphine tolerance. Morphine can activate inflammatory signaling pathways by directly binding to the TLR4/MD-2 complex on microglial membranes (Hutchinson et al., 2010; Lewis et al., 2010). In addition, morphine indirectly activate the TLR4 signaling pathway in microglia by inducing neurons to release damage-associated molecular patterns (DAMPs), such as high-mobility group box 1 (HMGB1) (Qian et al., 2020; Lin et al., 2023) and heat shock protein 70 (HSP70) (Qu et al., 2017). Chronic morphine administration increases the expression of TCF7L2 in microglia, which further upregulates TLR4 expression through transcriptional regulation (Chen et al., 2021). Upon activation, TLR4 induces the expression of myeloid differentiation primary response 88 (MyD88) in spinal cord tissue (Xu et al., 2014), which recruits the interleukin-1 receptor-associated kinase (IRAK) complex and activates the transforming growth factor β-activated kinase 1 (TAK1) signaling pathway, leading to the phosphorylation of IκB and mitogen-activated protein kinases (MAPKs). Phosphorylation of IκB promotes the nuclear translocation of NF-κB, while MAPK activation triggers the transcription factor AP-1 (Bai et al., 2014; Wang et al., 2021). Moreover, PKCε is a critical downstream target of TLR4. High doses of morphine activate the PKCε-Akt-ERK1/2 signaling cascade through a TLR4-dependent mechanism, enhancing the transcriptional activity of NF-κB (Merighi et al., 2013). These signaling pathways collectively promote the generation and release of pro-inflammatory cytokines, thereby exacerbating the inflammatory response. However, some studies have suggested that microglial activation in morphine tolerance may not be dependent on the TLR4 signaling pathway (Mattioli et al., 2014; Fukagawa et al., 2013), which may be attributed to differences in the mouse strains and animal models used.

In addition to TLR4, TLR2 is also involved in morphine-induced microglial activation (Zhang et al., 2011). During the development of morphine tolerance, TLR2 activates the NLRP3 inflammasome through the MyD88-dependent pathway, thereby mediating neuroinflammatory responses (Peng et al., 2023). TLR3 is an endosomal receptor that recognizes double-stranded RNA (dsRNA) and plays a unique role in chronic morphine tolerance. Morphine downregulates adenosine deaminase RNA-specific 1 (ADAR1) in neurons, leading to the accumulation of dsRNA. Subsequently, this accumulated dsRNA is taken up by microglia via exosomes, activating the TLR3-TRIF pathway and promoting the release of inflammatory mediators and exacerbating morphine tolerance (Wang B. et al., 2024).

Thus, multiple members of the TLR family may play a critical role in the development of morphine tolerance. Future studies should further explore the mechanisms underlying the involvement of these receptors in morphine tolerance to provide new insights for the development of targeted therapeutic strategies.

#### 4.1.3 P2X purinergic receptors (P2XRs)

P2XRs are ligand-gated ion channels that primarily respond to ATP. Among these receptors, P2X4R and P2X7R are involved in the development of morphine tolerance by modulating microglial activation. These two receptors exhibit significant differences in their sensitivity to ATP: P2X4R is activated at lower ATP concentrations (≤0.1 mM), whereas P2X7R typically requires higher ATP concentrations (≥1.0 mM) for activation (Asatryan et al., 2018). This concentration-dependent difference has been confirmed in human and mouse microglia.

Under physiological conditions, P2X7R typically remains in an inactive state. Chronic morphine exposure increases glutamate levels in the cerebrospinal fluid (Wen et al., 2004), triggering ATP release from spinal cord glial cells through an α-amino-3-hydroxy-5-methyl-4-isoxazolepropionic acid receptor (AMPAR)-mediated, Ca^2+^-dependent route (Liu G. J. et al., 2006). This creates a local microenvironment with elevated ATP concentrations, which activates P2X7R. Activation of P2X7R further promotes the release of ATP and glutamate (Suadicani et al., 2006), establishing a positive feedback loop. Furthermore, morphine can specifically phosphorylate the Y_382-384_ site in the C-terminal domain of P2X7R via the MOR-Src kinase signaling pathway, significantly enhancing P2X7R channel activity in microglia (Leduc-Pessah et al., 2017). Therefore, developing small molecule inhibitors targeting this phosphorylation site could offer a potential therapeutic strategy for morphine tolerance. Notably, gene knockout and pharmacological inhibition studies have shown that P2X7R primarily participates in the induction phase of morphine tolerance, rather than its maintenance phase (Wang H. et al., 2020).

P2X4R also plays a critical role in morphine tolerance. Intrathecal injection of P2X4R antisense oligonucleotides significantly delays the onset of morphine tolerance (Horvath et al., 2010). Moreover, TLR4 signaling may contribute to the upregulation of P2X4R in microglia. Specifically, morphine enhances the membrane trafficking of P2X4R by promoting TLR4-mediated endocytosis, thereby increasing the release of inflammatory factors such as IL-1β (Liang et al., 2016).

In summary, P2XRs interact with signaling pathways such as AMPAR and TLR4, establishing a feedback network that leads to an imbalance between neuronal excitation and inhibition. Targeted interventions on P2XRs can effectively disrupt the positive feedback loop between microglia and neurons, providing a novel therapeutic strategy for alleviating morphine tolerance in clinical settings.

#### 4.1.4 Receptor tyrosine kinases (RTKs)

RTKs are transmembrane receptors that regulate essential physiological and pathological processes, including cell proliferation, differentiation, migration, metabolism, and immune responses.

The epidermal growth factor receptor (EGFR) is involved in the development of morphine tolerance in various rodent models, including the cancer-induced bone pain rat model (Yang et al., 2021) and the spinal nerve ligation rat model (Puig et al., 2020). Studies have shown that gefitinib, a clinically approved EGFR inhibitor, effectively prevents and reverses morphine tolerance in rats (Puig et al., 2020). EGFR may play a role in the development of morphine tolerance by binding to its ligands, such as TNF-α, and activating ERK and other downstream signaling pathways.

Platelet-derived growth factor receptor β (PDGFRβ) is another important member of the RTK family. Studies have shown that inhibiting the PDGFRβ signaling pathway can reverse morphine-induced analgesic tolerance (Wang et al., 2012). Additionally, RTKs interact with members of the G protein-coupled receptor (GPCR) family, including MOR. Morphine acts on MOR to activate the c-Jun N-terminal kinase (JNK) signaling cascade, thereby promoting the phosphorylation of PDGFRβ (Li et al., 2020). The activated PDGFRβ then induces autophagy in GABAergic inhibitory interneurons in the dorsal horn of the spinal cord via its downstream p38 MAPK signaling pathway (Jia et al., 2021), which may facilitate central sensitization by reducing the release of inhibitory neurotransmitters.

In summary, RTKs such as EGFR and PDGFRβ play key roles in the development of morphine tolerance. The clinically approved EGFR inhibitor gefitinib could be translated into clinical strategies, offering a precise and efficient intervention for blocking tolerance.

### 4.2 Signaling pathways regulating microglial activation in morphine tolerance

#### 4.2.1 Mitogen-activated protein kinase (MAPK) signaling pathway

The MAPK family includes ERK, JNK, and p38 MAPK (Chen and Sommer, 2009), with p38 MAPK playing a pivotal role in the mechanism of morphine tolerance. After repeated intrathecal morphine administration in rats, the number of phosphorylated p38-immunoreactive cells in laminae I-IV of the spinal dorsal horn increases significantly by day 3 and remains elevated until at least day 7. Double-labelling immunohistochemistry revealed that the majority of these cells were microglia (Cui et al., 2006). Consistently, the microglial inhibitor minocycline significantly attenuates morphine tolerance by inhibiting p38 activity. In addition to TLR4, both P2X7R (Zhou et al., 2010) and PDGFRβ (Jia et al., 2021) can trigger downstream cascades, ultimately leading to p38 phosphorylation in microglia. In parallel, sustained morphine exposure potentiates microglial p38 signaling through several convergent mechanisms, including upregulation of the E3 ubiquitin ligase Pellino one in microglia (Wang L. et al., 2020), lysosomal release of cathepsin S (CTSS) (Xiao et al., 2019), and neuronal production of NO (Liu W. et al., 2006), along with the release of calcitonin gene-related peptide (CGRP) (Wang et al., 2010a; Wang et al., 2009; Wang et al., 2010b). Activated p38 subsequently enhances NF-κB transcriptional activity through phosphorylation of the p65 subunit, promoting the expression of IL-1β, TNF-α, and IL-6. This ultimately exacerbates neuroinflammation and accelerates morphine tolerance. Therefore, selective inhibition of the p38 MAPK pathway may represent a potential therapeutic target for mitigating morphine tolerance.

#### 4.2.2 NOD-like receptor protein 3 (NLRP3) signaling pathway

The NLRP3 inflammasome, composed of the receptor NLRP3, the adaptor protein ASC, and the inactive pro-caspase-1, serves as a core platform for amplifying inflammatory signals in the innate immune system. Morphine triggers its assembly through two primary pathways: first, morphine directly activates TLR4/TLR2, inducing a conformational change in NLRP3, leading to its oligomerization (Wang et al., 2021; Peng et al., 2023); second, morphine activates the P2X7R channel, causing K^+^ efflux, and the resulting low intracellular K^+^ concentration further stabilizes the NLRP3 oligomer (Wang H. et al., 2020). Additionally, morphine-induced mitochondrial dysfunction generates large amounts of reactive oxygen species (ROS) and promotes lysosomal rupture, releasing cathepsin B (CTSB) (Liu et al., 2020; Hayashi et al., 2014). These danger signals collectively function as a second stimulus, recruiting ASC and activating pro-caspase-1, ultimately leading to its cleavage. The activated caspase-1 processes pro-IL-1β and pro-IL-18 into their mature forms and rapidly releases them, further amplifying neuroinflammation, enhancing synaptic plasticity abnormalities, and thereby accelerating the development of morphine tolerance. Therefore, targeting the activity of the NLRP3 inflammasome may be a key strategy for mitigating morphine tolerance.

#### 4.2.3 AMP-activated protein kinase (AMPK) signaling pathway

AMPK is a cellular energy sensor that is activated when the AMP/ATP or ADP/ATP ratio increases. Recent studies have revealed that this “energy switch” also regulates neuroinflammation: its agonist, metformin, significantly delays morphine tolerance by inhibiting the excessive activation of microglia (Pan et al., 2016). In addition, suppressor of cytokine signaling 3 (SOCS3) serves as a negative regulator of microglial activation. Lidocaine enhances AMPK phosphorylation via a calcium-dependent protein kinase kinase β (CaMKKβ)-dependent pathway, upregulating SOCS3 expression, which in turn suppresses the release of IL-1β and TNF-α, alleviating morphine tolerance (Zhang et al., 2017). On the other hand, AMPK-mediated autophagy activation degrades DICER and AGO2, thereby blocking the maturation of miR-30a-5p, which upregulates SOCS3 and further suppresses inflammatory signaling (Wan et al., 2022). Therefore, the cross-regulatory role of AMPK in energy metabolism and inflammation may provide new perspectives for alleviating neuroinflammation and mitigating morphine tolerance.

**FIGURE 1:**
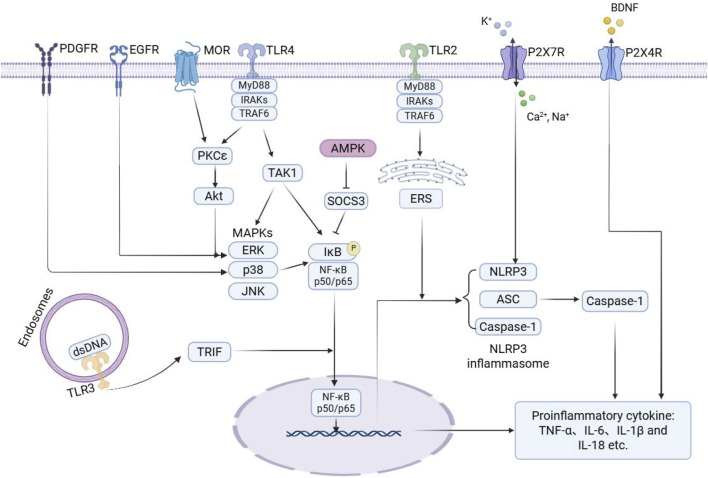
Schematic illustration of microglial signaling pathways associated with morphine tolerance. During the development of morphine tolerance, morphine activates key receptors on microglia, directly or indirectly, such as MOR, TLRs, P2XRs, and RTKs. This activation then triggers downstream signaling pathways, including MAPK, NF-κB, NLRP3, and AMPK. The activation of these pathways promotes the release of various pro-inflammatory mediators from microglia, thereby exacerbating neuroinflammation and contributing to the onset of morphine tolerance. Arrows indicate activation, and blunt-ended lines represent inhibition. PDGFR: platelet-derived growth factor receptor; EGFR: epidermal growth factor receptor; MOR: μ-opioid receptor; TLR: toll-like receptor; P2X7R: purinergic P2X7 receptor; P2X4R: purinergic P2X4 receptor; BDNF: brain-derived neurotrophic factor; MyD88: myeloid differentiation primary response 88; IRAKs: interleukin-1 receptor-associated kinases; TRAF6: TNF receptor-associated factor 6; PKCε: protein kinase C epsilon; Akt: protein kinase B; TAK1: transforming growth factor β-activated kinase 1; AMPK: AMP-activated protein kinase; SOCS3: suppressor of cytokine signaling 3; ERS: endoplasmic reticulum stress; MAPKs: mitogen-activated protein kinases; ERK: extracellular signal-regulated kinase; JNK: c-Jun N-terminal kinase; IκB: inhibitor of kappa B; NF-κB: nuclear factor kappa B; NLRP3: NOD-like receptor protein 3; ASC: apoptosis-associated speck-like protein containing a CARD; TRIF: TIR-domain containing adaptor inducing interferon-β; dsDNA: double-stranded DNA; TNF-α: tumor necrosis factor-α.

In conclusion, the receptors and signaling pathways discussed above play a crucial role in the progression of morphine tolerance by driving microglial activation and amplifying inflammatory responses. An overview of the microglial signaling pathways associated with morphine tolerance is shown in Figure 1.

### 4.3 Cytokines mediating interactions between microglia, astrocytes, and neurons

Upon activation of signaling pathways in microglia, a variety of cytokines are released, transmitting inflammatory signals to astrocytes and neurons in the microenvironment. This cytokine-mediated intercellular interaction plays a pivotal role in the development of morphine tolerance.

On one hand, prolonged morphine use activates microglia, prompting the release of pro-inflammatory cytokines such as TNF-α, IL-1α, IL-18, and complement C1q subcomponent subunit A (C1qA). These inflammatory mediators drive astrocytes to transition to a neurotoxic A1 phenotype. Complement C3 (C3) secreted by A1 astrocytes further activates microglia by binding to C3a receptors (C3aR), creating a positive feedback loop that exacerbates neuroinflammation (Peng et al., 2025). Meanwhile, D-serine released by activated astrocytes promotes morphine tolerance by activating N-methyl-D-aspartate receptor (NMDAR) on neurons and triggering the PKCγ signaling pathway (Chen et al., 2012).

On the other hand, cytokines such as IL-1β, TNF-α, and IL-6 secreted by microglia not only upregulate the expression and activity of neuronal MOR, forming a “neuroinflammation-MOR overactivation” positive feedback loop (Cuitavi et al., 2024), but also significantly interfere with glutamatergic neurotransmission. Studies have shown that the TNF-α inhibitor etanercept restores the functional expression of glutamate transporters GLT-1 and GLAST and downregulates the overactivated AMPA and NMDA receptor subunits (Shen et al., 2011). Additionally, microglia can release brain-derived neurotrophic factor (BDNF), which binds to the tropomyosin receptor kinase B (TrkB), activating downstream pathways such as MAPK/ERK and phosphatidylinositol 3-kinase (PI3K)/Akt. This upregulates the transcription and expression of vesicular glutamate transporter 2 (VGluT2), promoting glutamate release and increasing spinal cord neuronal excitability (He et al., 2022).

**FIGURE 2:**
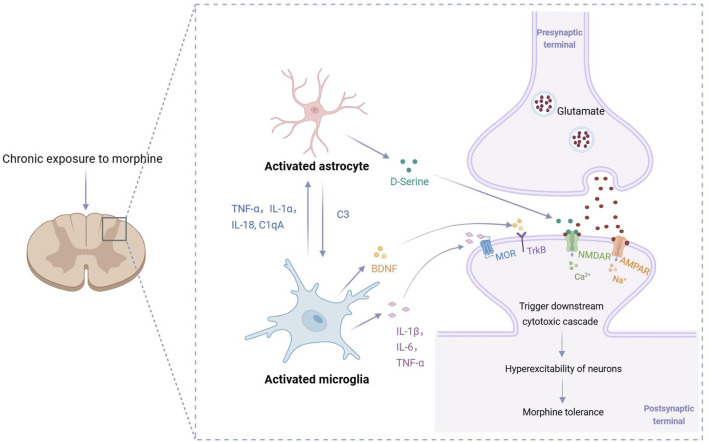
Cytokine-mediated mechanisms of morphine tolerance. Excessive activation of microglia releases cytokines that activate astrocytes, which in turn further activate microglia and amplify the inflammatory cascade. Additionally, the cytokines released by activated microglia and astrocytes (such as IL-1β, TNF-α, IL-6, D-serine and BDNF) collectively contribute to the excessive excitation of dorsal horn neurons in the spinal cord, thereby promoting the onset and progression of morphine tolerance. C1qA: complement C1q subcomponent subunit A; C3: complement C3; BDNF: brain-derived neurotrophic factor; MOR: μ-opioid receptor; TrkB: tropomyosin receptor kinase B; NMDAR: N-methyl-D-aspartate receptor; AMPAR: α-amino-3-hydroxy-5-methyl-4-isoxazolepropionic acid receptor.

In summary, microglia play a vital role in the development of morphine tolerance by mediating complex interactions among neuroimmune cells. The signaling network established between microglia, astrocytes, and neurons through cytokines is a key mechanism driving synaptic dysfunction, central sensitization, and the progression of morphine tolerance (Figure 2).

## 5 Phytochemicals alleviate morphine tolerance by modulating microglial activity

**FIGURE 3:**
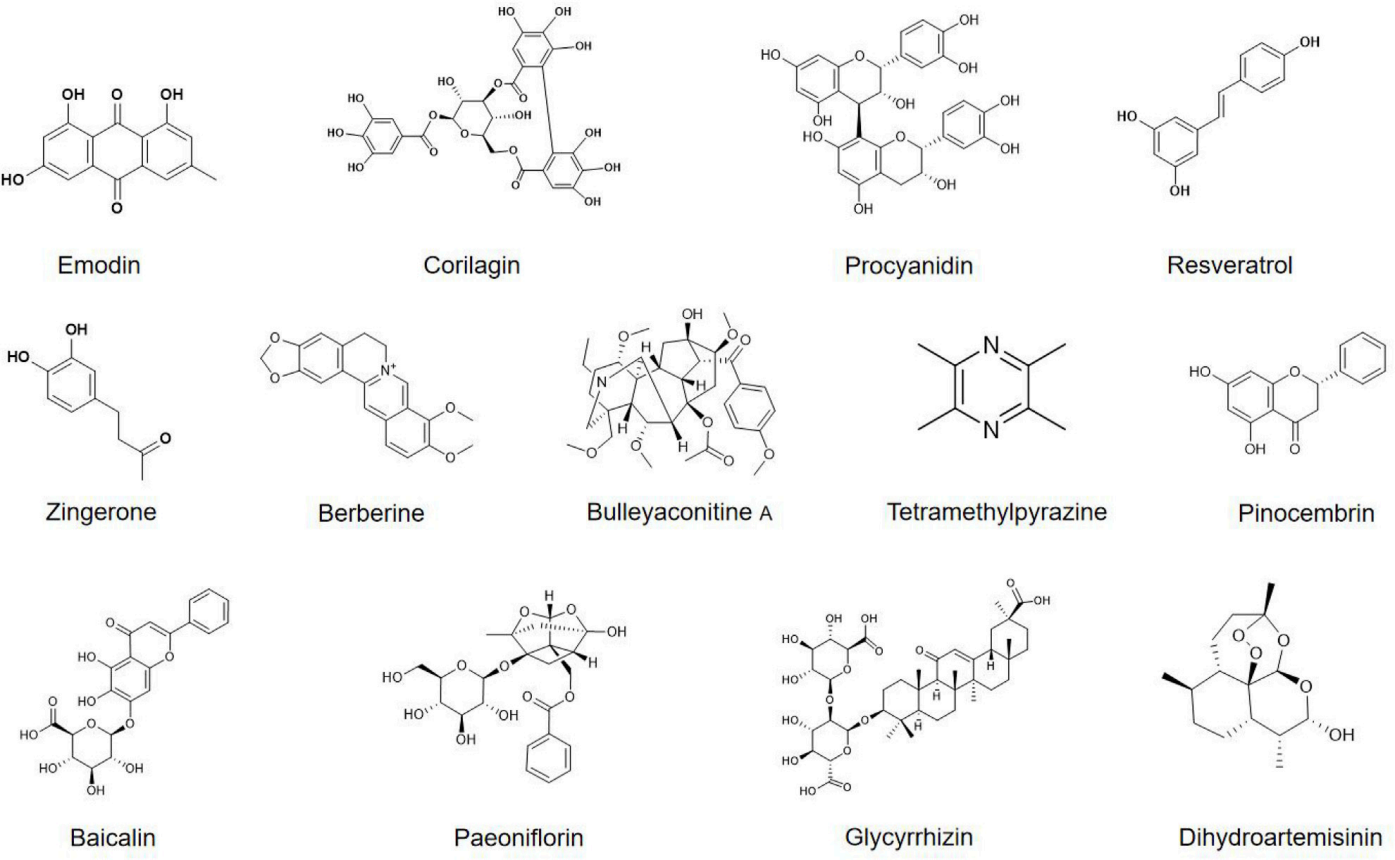
Chemical structures of phytochemicals that target microglia for alleviating morphine tolerance.

In recent years, phytochemicals have demonstrated significant potential in alleviating morphine tolerance due to their multi-target effects, high safety profile, and effective neuroimmune modulation. Several phytochemicals have been shown to delay the onset and progression of morphine tolerance by regulating microglial function (Table 1; Figure 3). Here we systematically overview these findings.

**TABLE 1:** Phytochemicals alleviate morphine tolerance by regulating microglia-mediated neuroinflammation in preclinical studies.

Category	Phytochemical	Source	Study type	Experimental model	Administration	Minimal active concentration	Biological target	Efficacy	Ref.
Anthraquinones	Emodin	*Rheum palmatum* L. [Polygonaceae; Rhei Radix et Rhizoma]*, Reynoutria japonica* Houtt. [Polygonaceae]	*In vitro*	BV2 cell	Emodin: 5, 10, and 20 μM, 18 h; morphine: 200 μM, 6 h	5 μM	Inhibition of TLR4/NF-κB/NLRP3 pathway	↑ SLC7A11, GPX4; ↓ TLR4, p-NF-κB p65, NLRP3, ASC, ACSL4, TFR1, TNF-α, IL-1β, IL-6, COX-2, iNOS	Li et al. (2024)
Phenolics	Corilagin	*Phyllanthus urinaria* L. [Phyllanthaceae]*, Phyllanthus emblica* L. [Phyllanthaceae; Phyllanthi Fructus]*, Phyllanthus niruri* L. [Phyllanthaceae; Phyllanthi niruri herba]	*In vitro*	BV2 cell	Corilagin: 0.1, 1, and 10 μM, 18 h; morphine: 200 μM, 6 h	0.1 μM	Inhibition of TLR2/ERS/NLRP3 pathway	↓ TLR2, NLRP3, ASC, cleaved-Caspase-1, GRP78, p-PERK, ATF4, CHOP, TNF-α, IL-1β, IL-6, COX-2, iNOS	Guan et al. (2023)
	Procyanidins	*Vitis vinifera* L. [Vitaceae]	*In vitro*	BV2 cell	Procyanidins: 100 μM, 15 min; morphine or LPS: 200 μM or 1 μg/mL, 12 h	100 μM	Reduction of ROS and inhibition of p38 MAPK/NF-κB/NLRP3 pathway	↓ p-p38 MAPK, NLRP3, ROS, Caspase-1, TNF-α, IL-1β, p-JNK, p-ERK, p-NR1, p-PKC	Cai et al. (2016)
			*In vivo*	CD-1 mice	Procyanidins: 20, 40, and 80 mg/kg, i.g., b.i.d.; 15 min later, morphine: 10 mg/kg, s.c. b.i.d., 7 days	20 mg/kg			
	Resveratrol	*Vitis vinifera* L. [Vitaceae], *Reynoutria japonica* Houtt. [Polygonaceae]	*In vitro*	BV2 cell	Resveratrol: 0.2, 2, and 20 μM, 15 min; morphine: 200 μM, 6 h	2 μM	Activation of AMPK and inhibition of p38MAPK/NF-κB pathway	↑ p-AMPK; ↓ p-p38 MAPK, TNF-α, IL-1β, IL-6, iNOS	Han et al. (2014)
			*In vivo*	Female CD-1 mice	Procyanidins: 40,80, and 160 mg/kg, i.p., q.d.; 15 min later, morphine: 10 mg/kg, s.c. q.d., 7 days	80 mg/kg			
	Zingerone (ZIN)	*Zingiber officinale* Roscoe [Zingiberaceae; Zingiberis Rhizoma]	*In vivo*	Male NMRI mice	ZIN: 100 mg/kg, p.o., q.d.; 30 min later, morphine: 10 mg/kg, i.p., q.d., 7 days	100 mg/kg	Inhibition of oxidative stress-mediated NLRP3 activation	↓ NLRP3, ASC, Caspase-1, IL-1β, TBARS, NO; ↑ TT, GPx	Molavinia et al. (2024)
Alkaloid	Berberine	*Coptis chinensis* Franch. [Ranunculaceae; Coptidis Rhizoma]	*In vitro*	BV2 cell	Berberine: 5,10, and 20 μM, 2 h; morphine: 200 μM, 24 h	20 μM	Inhibition of NF-κB pathway	↑ Bcl-2; ↓ TNF-α, IL-1β, Bax, Caspase-3	Shuai et al. (2025)
			*In vivo*	Female C57BL/6J mice	Berberine: 2.5, 5, and 10 mg/kg, i.p., q.d.; 30 min later, morphine: 10 mg/kg, s.c. q.d., 8 days	5 mg/kg			
	Bulleyaconitine A (BLA)	*Aconitum bulleyanum* Diels. [Ranunculaceae]	*In vivo*	L5 spinal nerve ligation in male Sprague-Dawley rats	BLA: 0.4 mg/kg, i.g., b.i.d.; 30 min later, morphine: 10 mg/kg, s.c., b.i.d., 10 days	0.4 mg/kg	Inhibition of microglia activation	↓ p-PKCγ	Mai et al. (2020)
	Tetramethylpyrazine (TMP)	*Ligusticum chuanxiong* Hort. [Apiaceae; Chuanxiong Rhizoma]	*In vivo*	Male ICR mice	TMP: 15, 30, and 60 mg/kg, i.p., q.d.; 15 min later, morphine: 10 mg/kg, s.c., q.d., 7 days	15 mg/kg	Inhibition of p38MAPK phosphorylation	↓ p-p38 MAPK, TNF-α, IL-1β-	Chen et al. (2015)
Flavonoids	Pinocembrin	*Pinus cembra* L. [Pinaceae], *Prunus avium* L. [Rosaceae], *Alpinia officinarum* Hance [Zingiberaceae; Alpiniae Officinari Rhizoma]	*In vitro*	BV2 cell	Pinocembrin: 50 μM, 6 h; co-administered with morphine: 200 μM	50 μM	Inhibition of STAT3 phosphorylation	↓ p-STAT3, TNF-α, IL-1β-	Han et al. (2022)
			*In vivo*	Male C57BL/6 mice	Pinocembrin: 25, 50, and 100 mg/kg, i.g., q.d.; 4 h later, morphine: 10 μg/μL/mouse, i.t., q.d., 7/14 days	25 mg/kg			
	Baicalin	*Scutellaria baicalensis* Georgi [Lamiaceae; Scutellariae Radix]	*In vitro*	BV2 cell	Baicalin: 1, 10, 100 μM, 30 min; morphine: 200 μM, 6 h	10 μM	Inhibition of p38MAPK phosphorylation	↓ p-p38 MAPK, TNF-α, IL-1β-	Song et al. (2014)
			*In* *vivo*	Female ICR mice	Baicalin: 20, 40, 60 μg/10 μL, i.t., q.d.; 10 min later, morphine: 10 mg/kg, s.c., q.d., 7 days	20 μg/10 μL			
Terpenoids	Paeoniflorin	*Paeonia lactiflora* Pall. [Paeoniaceae; Paeoniae Radix Alba/Paeoniae Radix Rubra]	*In vitro*	BV2 cell	Paeoniflorin: 1, 10, and 100 μM, 6 h; co-administered with morphine: 200 μM	1 μM	Inhibition of TLR4/p38MAPK/NF-κB pathway	↓ TLR4, p-p38 MAPK, p-NR1, TNF-α, IL-1β, IL-6	Jiang et al. (2015)
			*In vivo*	Male Sprague-Dawley rats and female CD-1 mice	Paeoniflorin: 20, 40, and 80 mg/kg, i.p., q.d.; 15 min later, morphine: 10 mg/kg, s.c., q.d., 7 days	40 mg/kg			
	Glycyrrhizin	*Glycyrrhiza glabra* L. [Fabaceae; Glycyrrhizae Radix et Rhizoma]	*In vivo*	Male Sprague–Dawley rats	Glycyrrhizin: 10, 20, and 40 μg/10 μL, i.t., q.d.; co-administered with morphine: 10 μg/10 μL, i.t., q.d., 6 days	10 μg/10 μL	Inhibition of HMGB1/TLR4/NF-κB pathway	↓ HMGB1, TLR4, NF-κB p65, TNF-α, IL-1β	Qian et al. (2020)
			*In vivo*	Male Institute of Cancer Research (ICR) mice and male Sprague-Dawley rats	Glycyrrhizin: 25, 50, and 100 mg/kg, i.g., q.d.; 15 min later, morphine: 10 μg/10 μL, i.t., q.d., 7 days	25 mg/kg	Inhibition of HMGB1/TLR4/NF-κB/NLRP3 pathway	↓ HMGB1, NF-κB p65, TNF-α, IL-1β	Lin et al. (2023)
	Dihydroartemisinin (DHA)	*Artemisia annua* L. [Asteraceae; Artemisiae Annue Herba]	*In vitro*	BV2 cell	DHA: 1,5,10, and 20 μM, 30 min; morphine: 200 μM, 24 h	5 μM	Increase the expression of miR-16 and inhibit TLR4/NF-κB pathway	↓ TLR4, TNF-α, IL-1β, IL-6	Guan et al. (2021)

↓: decrease or downregulate; ↑: increase or upregulate. SLC7A11: solute carrier family 7 member 11; GPX4: glutathione peroxidase 4; ACSL4: acyl-CoA, synthetase long-chain family member 4; TFR1: transferrin receptor 1; COX-2: cyclooxygenase-2; JKN: c-Jun N-terminal kinase; ERK: extracellular signal-regulated kinase; NR1: NMDA receptor subunit 1; TBARS: thiobarbituric acid reactive substances; TT: total thiol; GPx: glutathione peroxidase; i.g.: intragastric administration; s.c.: subcutaneous injection; i.p.: intraperitoneal injection; p.o.: oral administration; i.t.: intrathecal injection; q.d.: once a day; b.i.d.: twice a day.

### 5.1 Anthraquinones

Emodin (C_15_H_10_O_5_) is a natural anthraquinone primarily found in medicinal plants such as *Rheum palmatum* L. and *Reynoutria japonica* Houtt. It’s medicinal application history is over 2,000 years (Mitra et al., 2022). Modern pharmacological studies have shown that emodin possesses various bioactivities, including cardioprotective, antitumor, and anti-inflammatory effects (Zheng et al., 2021). Li et al. predicted a potential interaction between emodin and TLR4 through molecular docking and confirmed in BV2 microglial cells that emodin can block the TLR4/MyD88/NF-κB signaling pathway, inhibiting NLRP3 inflammasome activation, thereby reducing morphine-induced neuroinflammation (Li et al., 2024). However, these findings at the cellular level still need further validation in animal models.

### 5.2 Phenolics

Corilagin (C_27_H_22_O_18_) is a water-soluble natural tannin predominantly found in plants of the *Phyllanthus* genus, such as *Phyllanthus urinaria* L.*, Phyllanthus emblica* L.*, and Phyllanthus niruri* L. Modern pharmacological studies have confirmed its antioxidant, anti-inflammatory, and antitumor activities (Wang et al., 2023). Guan et al. used molecular docking to predict that corilagin binds to TLR2 with an affinity of −5.4 kcal/mol (moderate binding). In BV2 cells, corilagin downregulates TLR2, thereby blocking endoplasmic reticulum stress (ERS) and inhibiting NLRP3 inflammasome activation, ultimately attenuating morphine-induced microglial overactivation (Guan et al., 2023). However, these cellular-level findings require further validation in animal models to confirm their translational relevance.

Procyanidin (C_30_H_26_O_13_) is a natural polyphenol widely distributed in plants. It is a condensed tannin formed by polymerization of flavan-3-ol monomers, with *Vitis vinifera* L. being the most abundant source (Wang Y. et al., 2024). Procyanidin possesses potent antioxidant and anti-inflammatory activities and a high safety profile, with a maximum tolerated dose of 2,500 mg/day for adults (Sano, 2017), making it widely used as a dietary supplement. In mouse studies, Cai and colleagues demonstrated that procyanidin can alleviate the development of chronic morphine tolerance. The underlying mechanism involves the inhibition of p38 MAPK phosphorylation, blockade of p65 NF-κB nuclear translocation, and subsequent suppression of NLRP3 inflammasome activation, thereby reducing neuroinflammation. Additionally, procyanidin significantly decreases morphine-induced ROS levels (Cai et al., 2016). These findings suggest that procyanidin holds strong therapeutic potential for mitigating morphine tolerance through modulation of oxidative stress and inflammatory signaling pathways.

Resveratrol (C_14_H_12_O_3_) is a natural polyphenol found abundantly in *Vitis vinifera* L. and *Reynoutria japonica* Houtt. It exhibits antioxidant, anti-inflammatory, anti-cancer, and neuroprotective effects (Radeva and Yoncheva, 2025). Animal studies have shown that resveratrol significantly attenuates chronic morphine tolerance (Tsai et al., 2016; Tsai et al., 2012). Mechanistically, resveratrol activates AMPK, suppresses the downstream p38 MAPK/NF-κB signaling pathway, and thereby reduces microglia-mediated neuroinflammation, delaying the development of morphine tolerance (Han et al., 2014).

Zingerone (ZIN, C_11_H_14_O_3_) is a natural phenolic primarily extracted from *Zingiber officinale* Roscoe. It exhibits antioxidant, anti-inflammatory, antibacterial, and immunomodulatory activities (Rashpa et al., 2025). ZIN has a favorable safety profile, with an oral LD_50_ of 657.55 mg/kg in mice. In animal studies, co-administration of ZIN with morphine significantly prolongs the thermal nociceptive latency, enhancing and sustaining the analgesic effect of morphine. The underlying mechanism may involve the inhibition of NLRP3 inflammasome activation, which is induced by oxidative stress (Molavinia et al., 2024).

### 5.3 Alkaloids

Berberine (C_20_H_18_NO_4_^+^) is a typical isoquinoline alkaloid primarily extracted from *Coptis chinensis* Franch. It exhibits a variety of pharmacological activities, including antioxidant, anti-inflammatory, anticancer, immunomodulatory, and antimicrobial activities. In recent years, several *in vivo* studies have demonstrated that berberine effectively inhibits the development of analgesic tolerance induced by prolonged morphine administration (Shuai et al., 2023; Yoo et al., 2006). Han et al. further elucidated the underlying mechanism through *in vitro* experiments, showing that berberine inhibits morphine-induced activation of BV2 cells, suppresses NF-κB signaling to reduce the release of pro-inflammatory cytokines, and mitigates morphine-induced apoptosis by regulating apoptosis-related proteins, including upregulating Bcl-2 and downregulating Bax and Caspase-3 (Shuai et al., 2025). These findings provide important insights into the pharmacological mechanisms of berberine in attenuating morphine tolerance.

Bulleyaconitine A (BLA, C_35_H_49_NO_9_) is a C19-diterpenoid alkaloid isolated from *Aconitum bulleyanum* Diels., known for its potent analgesic activity (Zhao et al., 2021). Since 1985, BLA has been widely used in China for the treatment of chronic pain. Multiple studies have demonstrated that oral administration of BLA effectively alleviates various types of chronic pain, with favorable safety and tolerability profiles (Xie et al., 2018). In a rat model of neuropathic pain induced by L5 spinal nerve ligation (L5-SNL), BLA delayed the development of morphine tolerance. The underlying mechanism may involve the inhibition of PKCγ phosphorylation in spinal dorsal horn neurons, as well as the attenuation of abnormal activation of microglia and astrocytes (Mai et al., 2020). However, the precise mechanisms by which BLA inhibits microglia-mediated inflammatory responses remain to be fully elucidated.

Tetramethylpyrazine (TMP, C_8_H_12_N_2_) is the principal alkaloid isolated from the rhizome of *Ligusticum chuanxiong* Hort., and has been documented in the *Shen Nong Ben Cao Jing* for its ability to activate blood circulation and relieve pain (Lin et al., 2022). Modern studies have shown that TMP not only exerts anti-inflammatory and antioxidant effects but also crosses the blood-brain barrier, exerting pharmacological actions within the central nervous system. Chen et al. have suggested that TMP may delay the onset of morphine tolerance by inhibiting the phosphorylation of p38 MAPK in microglial cells, thereby reducing the expression of pro-inflammatory cytokines such as TNF-α and IL-1β (Chen et al., 2015).

### 5.4 Flavonoids

Pinocembrin (C_15_H_12_O_4_) is a naturally occurring flavonoid found in plants such as *Pinus cembra* L., *Prunus avium* L., and *Alpinia officinarum* Hance. It readily crosses the blood–brain barrier and exhibits broad pharmacological activities, including antibacterial, anti-inflammatory, antioxidant, and anticancer activities (Gong et al., 2020). In mouse models of chronic morphine exposure, Han et al. demonstrated that pinocembrin not only prevents the development of morphine tolerance but also reverses established tolerance. The underlying mechanism appears to involve the inhibition of signal transducer and activator of transcription 3 (STAT3) phosphorylation in microglia, leading to the downregulation of pro-inflammatory cytokines such as TNF-α and IL-1β, attenuation of neuroinflammation, and restoration of morphine analgesic efficacy (Han et al., 2022).

Baicalin (C_21_H_18_O_11_) is a flavonoid isolated from the dried roots of *Scutellaria baicalensis* Georgi. It exhibits a wide range of pharmacological activities, including antibacterial, anti-inflammatory, antioxidant, sedative, cardioprotective, and neuroprotective effects (Wen et al., 2025). Song et al. found that baicalin significantly alleviates morphine tolerance. The underlying mechanism may involve the inhibition of aberrant p38 MAPK signaling pathway activation, thereby reducing morphine-induced microglial overactivation (Song et al., 2014).

### 5.5 Terpenoids

Paeoniflorin (C_23_H_28_O_11_) is a water-soluble monoterpene glycoside extracted from the roots of *Paeonia lactiflora* Pall., known for its antioxidant, anti-inflammatory, analgesic, and neuroprotective effects (Hong et al., 2022). In a mouse model of morphine tolerance, Jiang et al. demonstrated that paeoniflorin significantly attenuates the development of morphine tolerance. The underlying mechanisms include downregulation of TLR4 expression, inhibition of p38 MAPK phosphorylation, and blockade of NF-κB nuclear translocation, collectively reducing the release of pro-inflammatory cytokines such as TNF-α, IL-1β, and IL-6 (Jiang et al., 2015).

*Glycyrrhiza glabra* L. is a cornerstone medicinal plant in traditional Chinese medicine with a long clinical history. The *Shen Nong Ben Cao Jing* documented its ability to tonify the heart and spleen and to harmonize the flow of Qi (vital energy), and it has been traditionally used for pain relief, expectoration, and cough suppression (Wahab et al., 2021). Glycyrrhizin (C_42_H_62_O_16_), the principal active metabolite of its root, has been shown to attenuate morphine tolerance in animal models. Chronic morphine administration induces spinal neurons to release HMGB1, which activates the microglial TLR4/NF-κB/NLRP3 signaling axis, thereby fostering tolerance. Glycyrrhizin inhibits HMGB1, blocking this pathway, reducing neuroinflammation, and restoring the analgesic efficacy of morphine (Qian et al., 2020; Lin et al., 2023).

Dihydroartemisinin (DHA, C_15_H_24_O_5_) is a reduced derivative of artemisinin isolated from *Artemisia annua* L. and belongs to the sesquiterpene lactone family. Although widely used as a first-line antimalarial, DHA also exhibits immunomodulatory, antitumor, and anti-inflammatory activities (Yu et al., 2021). Guan et al. demonstrated *in vitro* that DHA upregulates miRNA-16, thereby suppressing overactivation of the TLR4/NF-κB pathway and attenuating morphine-induced BV2 microglial inflammation, which in turn attenuates the development of morphine tolerance (Guan et al., 2021). However, these cellular findings still need further validation in animal models.

## 6 Clinical translational challenges and strategies of phytochemicals in treating morphine tolerance

Although phytochemicals demonstrate significant therapeutic potential in alleviating morphine tolerance, several challenges remain in translating them into clinical practice.

### 6.1 Optimization of pharmacokinetics and nanocarrier delivery

Phytochemicals often face significant pharmacokinetic challenges, such as poor solubility, rapid metabolism, and low bioavailability, which severely limit their clinical application. For example, emodin is rapidly metabolized into its glucoside form in the body, losing its bioactivity and significantly reducing its bioavailability, thereby restricting its clinical use (Sharifi-Rad et al., 2022). Similarly, DHA, due to its unique structure, exhibits poor water solubility and low bioavailability, often requiring multiple injections to achieve the desired therapeutic effect (Dai et al., 2021). The advent of nanoparticle delivery systems provides a practical solution to these issues. Technologies such as liposomes, nanoparticles, polymeric micelles, and nanogels can effectively improve the *in vivo* distribution properties of drugs and enhance targeted delivery efficiency. For instance, recent preclinical studies have shown that nanoformulated berberine not only retained its antitumor activity comparable to conventional therapies but also reduced the required dose to one-tenth of the traditional dosage, significantly reducing gastrointestinal side effects (Sun et al., 2024). Additionally, several studies have demonstrated that nanocarrier technologies can effectively reduce the toxicity of phytochemicals (Ai et al., 2025; Zhao et al., 2020). Therefore, the continued development and refinement of nanoparticle-based delivery systems are crucial for enhancing the clinical utility of phytochemicals in the treatment of morphine tolerance.

### 6.2 Advancement of clinical evidence and translational research

Clinical research on phytochemicals for the treatment of morphine tolerance remains scarce, lacking large-scale, high-quality clinical trial data. This has become a major bottleneck for their clinical translation, as their efficacy and safety have not been fully validated. Fortunately, some phytochemicals have gradually entered clinical trial phases. For example, a Phase I clinical trial involving 58 healthy participants demonstrated that intravenous administration of glucosides (maximum daily dose of 120 mg) have acceptable safety and tolerability (Cao et al., 2015). Glycyrrhizin, the main active metabolite of *Glycyrrhiza glabra* L., has been recognized for its safety by the Joint Expert Committee on Food Additives of the World Health Organization (Huan et al., 2021). In addition, some phytochemicals have entered the market in the form of dietary supplements. For example, resveratrol has long been considered to have potential health benefits, with approximately 6,000 participants from different age groups (including children and the elderly) involved in related trials. Although resveratrol has demonstrated good tolerability at doses up to 1 g/day, standardized dosing regimens for specific diseases or therapeutic targets have yet to be established (Brown et al., 2024). Therefore, to promote the clinical application of phytochemicals in alleviating morphine tolerance, large-scale, multi-center, randomized controlled clinical trials are warranted. Through rigorous study designs and sound statistical analysis methods, their efficacy and safety should be evaluated systematically, reliable clinical evidence could provid a solid foundation for their eventual clinical application.

### 6.3 Enhancement of safety and risk mitigation

Although phytochemicals are widely studied and appreciated for their natural origin and relatively mild side effects, their potential toxicity risks cannot be ignored. For instance, BLA, a potent analgesic, is widely used in the treatment of conditions such as rheumatoid arthritis, osteoarthritis, and lumbosacral pain. However, its application is often accompanied by adverse reactions such as nausea, palpitations, and rashes (Li et al., 2022). Similarly, although emodin has a wide range of pharmacological activities and is considered a promising phytochemical, studies have shown that it may induce hepatocyte apoptosis, cause renal damage, and lead to reproductive dysfunction (Chen et al., 2024). Therefore, before clinical advancement of phytochemicals, comprehensive and systematic toxicological studies must be conducted. These should include assessments of acute toxicity, chronic toxicity, genotoxicity, and reproductive toxicity, to determine safe dosage ranges and provide reliable scientific evidence for their safe clinical use.

### 6.4 Improvement of standardization and quality assurance

Standardization and stringent quality control are the cornerstones for the sustainable clinical application of phytochemicals. Given the diverse sources and complex compositions of phytochemicals, it is crucial to establish systematic quality standards and standardized dosing regimens. Modern analytical techniques such as high-performance liquid chromatography (HPLC) and mass spectrometry should be employed to comprehensively characterize candidate compounds and accurately identify active constituents, thereby facilitating the optimization of their bioavailability and dosing strategies. Additionally, it is essential to develop regulatory frameworks that comply with international standards, clearly defining the requirements for the production, quality assessment, and clinical use of phytochemicals to ensure their safety and efficacy.

## 7 Conclusions and prospects

Currently, there is still no fully effective treatment for morphine tolerance in clinical practice, prompting researchers to investigate phytochemicals as potential therapeutic strategies. Morphine tolerance involves is driven by a complex interplay of mechanisms, including neuroinflammation, aberrant microglial activation, and alterations in neural plasticity. Due to their multi-target and multi-pathway regulatory properties, phytochemicals align well with the “single drug-multiple targets” therapeutic concept, and thus demonstrate unique therapeutic potential.

This review highlights that phytochemicals can effectively alleviate morphine tolerance by inhibiting microglial activation and the subsequent neuroinflammatory response. Notably, different types of phytochemicals may target similar signaling pathways, with TLRs and their downstream NF-κB and MAPK pathways being the most commonly regulated targets. Nevertheless, further research is required to comprehensively elucidate the multi-target mechanisms of phytochemicals, which will provide a more robust theoretical foundation for their clinical application in the treatment of morphine tolerance.

Through a systematic analysis of the current literature, we identified key limitations in the existing research: (1) Most studies use male animal models, without adequately accounting for sex differences in drug efficacy. Future studies should incorporate gender factors to assess the therapeutic potential of phytochemicals more comprehensively. (2) Pain models not mimicking clinical conditions well enough. It is necessary to establish pain models that more closely resemble clinical conditions in order to enhance the clinical translation value of the findings. (3) Phytochemicals commonly face challenges such as low bioavailability, poor pharmacokinetic properties, potential toxicity, and unclear drug interactions. Addressing these challenges is crucial for advancing their clinical application.

Encouragingly, the rapid development of artificial intelligence technologies has led to significant progress in, receptor-ligand interaction prediction, particularly through molecular docking approaches. These developments offer new avenues for investigating the structure-activity relationships of phytochemicals and for designing efficient, low-toxicity derivatives. Moving forward, research should not only deepen our understanding of the mechanisms underlying phytochemical actions but also address key technological bottlenecks in clinical translation, thereby promoting more precise, standardized, and practical applications in the treatment of morphine tolerance.
